# Reservoir Cannulas for Pediatric Oxygen Therapy: A Proof-of-Concept Study

**DOI:** 10.1155/2016/9214389

**Published:** 2016-11-23

**Authors:** Grace Wu, Alec Wollen, Robert M. DiBlasi, Stephen Himley, Eugene Saxon, Glenn Austin, Jaclyn Delarosa, Rasa Izadnegahdar, Amy Sarah Ginsburg, Darin Zehrung

**Affiliations:** ^1^Department of Biomedical Engineering, Boston University, One Silber Way, Boston, MA 02215, USA; ^2^PATH, P.O. Box 900922, Seattle, WA 98109, USA; ^3^Seattle Children's Hospital, P.O. Box 5371, Seattle, WA 98145, USA; ^4^Bill & Melinda Gates Foundation, P.O. Box 23350, Seattle, WA 98102, USA; ^5^Save the Children, 501 Kings Highway East, Suite 400, Fairfield, CT 06825, USA

## Abstract

Hypoxemia is a complication of pneumonia—the leading infectious cause of death in children worldwide. Treatment generally requires oxygen-enriched air, but access in low-resource settings is expensive and unreliable. We explored use of reservoir cannulas (RCs), which yield oxygen savings in adults but have not been examined in children. Toddler, small child, and adolescent breathing profiles were simulated with artificial lung and airway models. An oxygen concentrator provided flow rates of 0 to 5 L/min via a standard nasal cannula (NC) or RC, and delivered oxygen fraction (FdO_2_) was measured. The oxygen savings ratio (SR) and absolute flow savings (AFS) were calculated, comparing NC and RC. We demonstrated proof-of-concept that pendant RCs could conserve oxygen during pediatric therapy. SR mean and standard deviation were 1.1 ± 0.2 to 1.4 ± 0.4, 1.1 ± 0.1 to 1.7 ± 0.3, and 1.3 ± 0.1 to 2.4 ± 0.3 for toddler, small child, and adolescent models, respectively. Maximum AFS observed were 0.3 ± 0.3, 0.2 ± 0.1, and 1.4 ± 0.3 L/min for the same models. RCs have the potential to reduce oxygen consumption during treatment of hypoxemia in children; however, further evaluation of products is needed, followed by clinical analysis in patients.

## 1. Introduction

Pneumonia is the leading infectious cause of death in children worldwide, accounting for 15% of all deaths in those under five years old. Pneumonia killed an estimated 920,136 children in this age group in 2015 [1]. Hypoxemia—lack of oxygen in the blood—is a major complication of pneumonia and strongly correlates with mortality in children [2–4]. Prevalence of hypoxemia in this population is estimated at 13% [4]. Hypoxemia can be treated with oxygen, a cost-effective and simple drug to administer, but effective treatment of hypoxic pediatric pneumonia requires a steady supply of oxygen.

The burden of pneumonia is highest in developing countries, yet oxygen sources are not always available or reliable in their health facilities [5–7]. In a survey of 590 health centers and hospitals across 22 low- and middle-income countries, only 42% reported that oxygen was always available for anesthetic care, whereas 35% had no source [8]. Another evaluation estimated that improving oxygen facilities could avert approximately 122,000 pediatric deaths from pneumonia per year [9]. The remaining facilities sometimes had oxygen available. Broken equipment, intermittent power, inadequate procurement resources, or missing accessories are probable causes of limited oxygen supplies [7, 10]. As per World Health Organization (WHO) guidelines and estimates from previous studies, recommended treatment involves administering oxygen for between 2 and 5 days at a continuous flow rate of up to 2 L/min via nasal catheter or nasal prongs [11, 12]. This amounts to 5,760 L to 14,400 L of oxygen per pediatric patient. The oxygen supply of hospitals may need to accommodate treating multiple pneumonia patients simultaneously, especially during the wet season [13].

The cost of oxygen supply technologies can be prohibitive. Central oxygen supplies—piped oxygen systems—are often too expensive to install and maintain, so facilities may rely on oxygen cylinders or oxygen concentrators. Oxygen cylinders must be replenished either via a delivery service or by on-site tank filling, both of which can be unreliable and expensive, especially in low-resource settings [14, 15]. In a study in Gambia, facilities that stocked oxygen cylinders were frequently hampered by transportation and other logistical issues and incurred significant costs; for example, a 6,000 L cylinder, enough to treat one child at 2 L/min for 2 days, costs US$38 [16]. Oxygen concentrators, at US$400 to US$1,000 retail price, may not be affordable for many facilities, especially primary health centers. These machines also depend on continuous power and maintenance, which are frequently unavailable at the facility [3].

Overall, there is an immediate need for affordable, cost-effective, and simple methods to improve oxygen availability in low-resource settings. Oxygen conservation methods could help address these needs by prolonging available oxygen resources. One potentially useful technology is the reservoir cannula (RC), which is similar to a standard nasal cannula (NC). Both deliver oxygen from the source to the patient, but an RC provides an additional concentrated oxygen bolus upon inspiration by collecting oxygen from the continuous flow between breaths with an inline 20 mL compliant reservoir [17]. As a result, the patient receives a higher delivered oxygen fraction (FdO_2_) from an RC than an NC, for a given flow rate. Manufacturer specifications state that an RC could conserve up to four times the volume of oxygen that would be needed if an NC were used (oxygen savings ratio [SR] of 4) [18]. For example, a 0.5 L/min flow using an RC would achieve the same oxygen saturation as a 2.0 L/min flow from a standard NC. In spite of significant oxygen conservation savings demonstrated in adults, we have found no reports on the development and use of RCs for pediatric patients.

The purpose of this study was to develop a method to assess the potential of RCs for pediatric patients, using an artificial test lung. We worked with upper airway models to simulate various patient profiles and measured the resulting FdO_2_ from existing RC products. We hypothesized that RCs could conserve oxygen and provide oxygen savings when compared with standard NCs in pediatric patients and that this framework can be applied to assess more complex breathing patterns in children.

## 2. Materials and Methods

### 2.1. Equipment

A variety of oxygen therapy devices and accessories were used during the cannula testing. This included adult and pediatric NCs (Salter Labs #1600-7, Lake Forest, IL, USA; Teleflex Hudson RCI #1871, Morrisville, NC, USA); oxygen tubing (Invacare #MS4107, Elyria, OH, USA); an AirSep Visionaire 5 oxygen concentrator (Chart Industries #AS098-4, Garfield Heights, OH, USA); and pendant Oxymizer RC (Chad Therapeutics #P-224, Lehigh Acres, FL, USA). Pediatric oxygen flowmeters were also used to provide finer flow control (Dwyer Instruments Inc. OMA-1 and OMA-2, Michigan City, IN, USA), and a flow analyzer (BC Group International, Inc. Flow Analyzer PFC-3000A, St. Charles, MO, USA) measured flow and oxygen percentage from the concentrator. Breathing profiles were created and simulated using a test lung (Ingmar Medical ASL 5000, Pittsburgh, PA, USA).

Toddler, small child, and adolescent airway models of ages 17 months, 5 years (Trudell Medical International, London, ON, Canada), and 17 years were used in this study. The adolescent model was produced from computed tomography scan data, verified by radiologists at Seattle Children's Hospital, and 3D printed in-house (Stratasys Objet30 Pro with VeroBlack resin, Eden Prairie, MN, USA). All models were integrated with standard 22 mm ventilator fitting. A two-way nonrebreathing valve (Hans Rudolph #1400, Shawnee, KS, USA) also was fitted onto the lung to allow only inspiration from the airway model, while venting expiration to the room.

### 2.2. Equipment Setup

**Figure 1:**
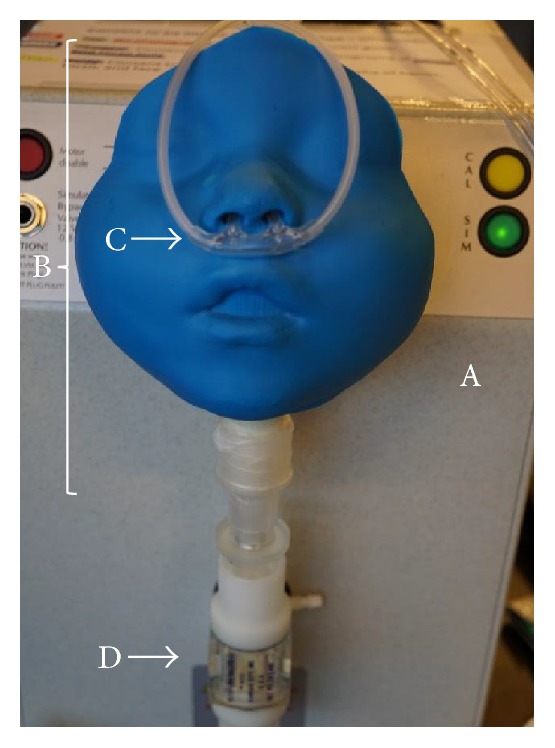
Equipment setup. (A) Test lung, (B) toddler airway model, (C) pediatric cannula, and (D) two-way valve.

**Figure 2:**
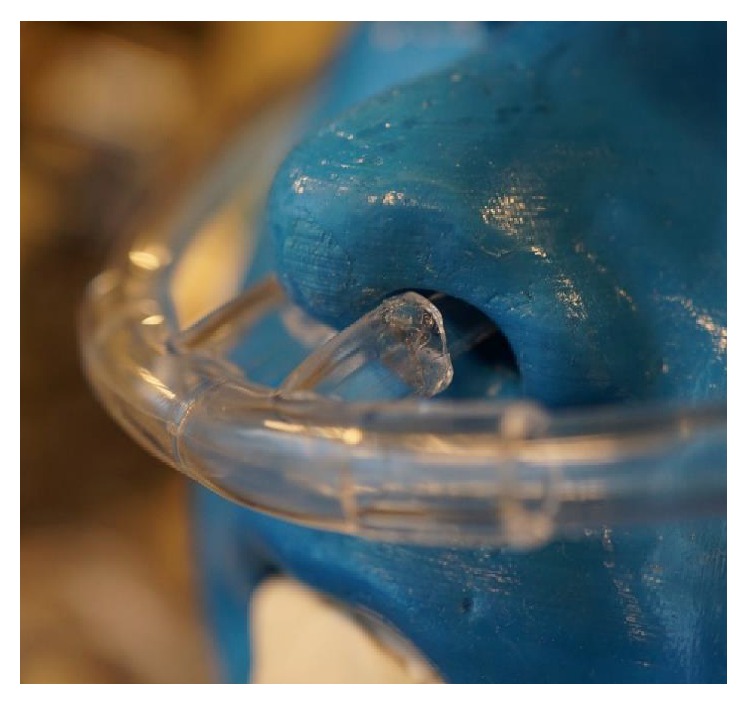
Model of child's airway with modified reservoir cannula.

The airway model, valve, cannula, and oxygen concentrator were set up with the test lung as shown in Figures 1 and 2. The test lung continuously measured FdO_2_ levels in the breathing chamber, which were recorded by the manufacturer's software. FdO_2_ measurements were recorded by averaging values of the last 10 breaths after running the breathing profile for 5 minutes, so that fluctuations were no more than ±1%. This was done with flow rates between 0.1 and 3 L/min for toddler and small child models and up to 5 L/min for the adolescent model. In addition, FdO_2_ values were normalized before recording to correct for 100% input oxygen delivery based on the calibration and measurement of concentrator output purity (89%–94%) by the flow analyzer.

**Figure 3:**
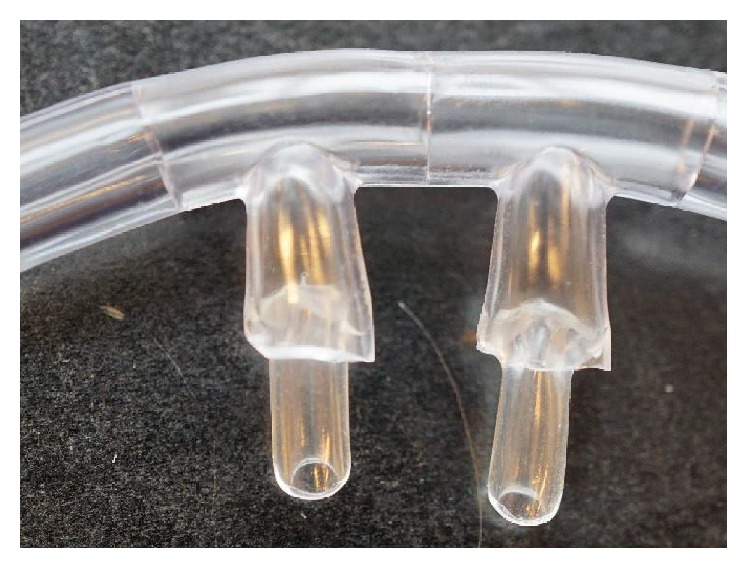
Modified reservoir cannula. Small nasal prongs were attached to the adult-sized prongs to accommodate the nares of the toddler and small child airway models.

Since the RC is available only in an adult size, the prongs were modified to better fit the toddler and small child models. Nasal prongs from pediatric NCs were cut off and inserted into the bore of the RC prongs. Silicone grease (Dow Corning High Vacuum Grease 14-635-5D, Midland, MI, USA) was added around the periphery of the prong insert to ensure a good seal (Figure 3). Adult NCs and RCs were used without modification for the adolescent model. Fit ratios, or the ratio of cross-sectional area of the nasal prong to the naris opening, were kept as close to 50% as possible, given the NC available. The fit ratios ranged from 35% to 70% as measured with calipers for all models tested.

**Figure 4:**
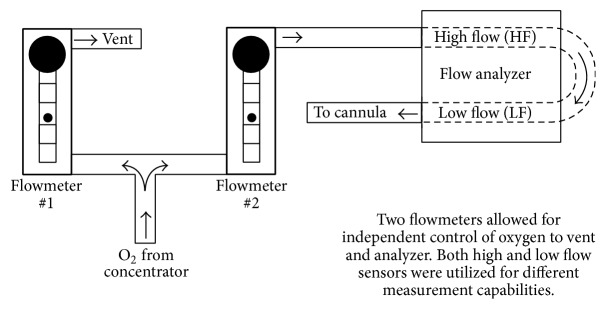
Setup of external flowmeters and flow analyzer.

A double-pediatric flow meter and flow analyzer arrangement was used for providing fine flow control to the cannula with resolution of 0.01 L/min and 0.1% oxygen (Figure 4). Concentrator output gas was allowed to vent, ensuring the concentrator stayed at a constant flow rate throughout the test to reduce oxygen concentration variation. Output flow was routed serially through the high-flow (HF) and low-flow (LF) circuits of the flow analyzer in order to measure HF oxygen concentration and high-precision LF flow rates delivered to the cannula. The LF flowmeter was not pressure compensated, so HF pressure readings were recorded to calculate compensated flows from the measured LF values.

### 2.3. Test Lung Settings

Three test lung profiles were used to simulate healthy adolescent, small child, and toddler patients matched with the available physical models used (Table 1). Pediatric profiles can vary enormously depending on age, weight, and height [19], as well as disease conditions (e.g., normal versus hypoxic pneumonia) [20–22]. We selected nominal patient settings based on several references [23–25].

### 2.4. Oxygen Savings Calculations

FdO_2_ measurements were recorded with the concentrator set at flow rates between 0 and 5 L/min, using both the NC and RC. The resulting FdO_2_ versus flow rate curves were used to estimate the oxygen savings. The oxygen savings ratio (SR) was defined as the ratio of two flow rates:

(1) $$\begin{array}{c} SR=\frac{Q_{NC}}{Q_{RC}}\;|\;{FdO}_{2,NC}={FdO}_{2,RC}. \end{array}$$

*Q*_NC_ and *Q*_RC_ were the flow rates delivered by the NC and RC, respectively, and FdO_2,NC_ and FdO_2,RC_ were the FdO_2_ levels delivered by an NC and an RC, respectively. *Q*_RC_ was computed through linear interpolation of two RC data points (RC1 and RC2):

(2) QRC=QRC1+FdO2,NC−FdO2,RC1∗QRC2−QRC1FdO2,RC2−FdO2,RC1.

Absolute oxygen flow rate savings (AFS) were defined similarly:

(3) $$\begin{array}{c} \text{AFS}=Q_{NC}-Q_{RC}\;|\;{FdO}_{2,NC}={FdO}_{2,RC}. \end{array}$$

Thus, SR was the ratio between the flow rate delivered by NC and the flow rate needed by RC to achieve the same FdO_2_. Theoretically, oxygen conservation is defined as an SR ratio greater than 1. AFS were the absolute difference in required flows between RC and NC to achieve the same FdO_2_, measured in L/min.

All data are presented as the mean ± one standard deviation of three readings (*n* = 3).

## 3. Results

### 3.1. Adolescent Results

**Figure 5:**
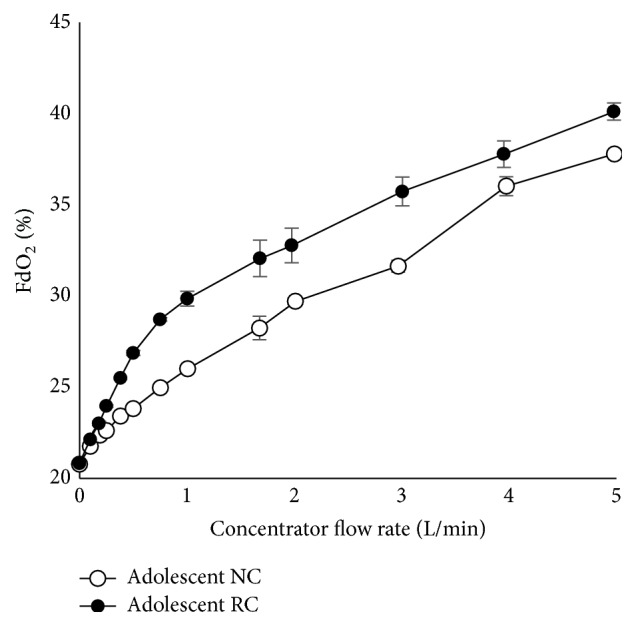
Performance test of reservoir cannula (RC) and nasal cannula (NC) on the adolescent lung model.

On adolescent lung settings, the unmodified RC conserved oxygen at all flow rates, with the greatest savings at approximately 1.5 L/min NC flow (Figure 5).

### 3.2. Small Child and Toddler Results

**Figure 6:**
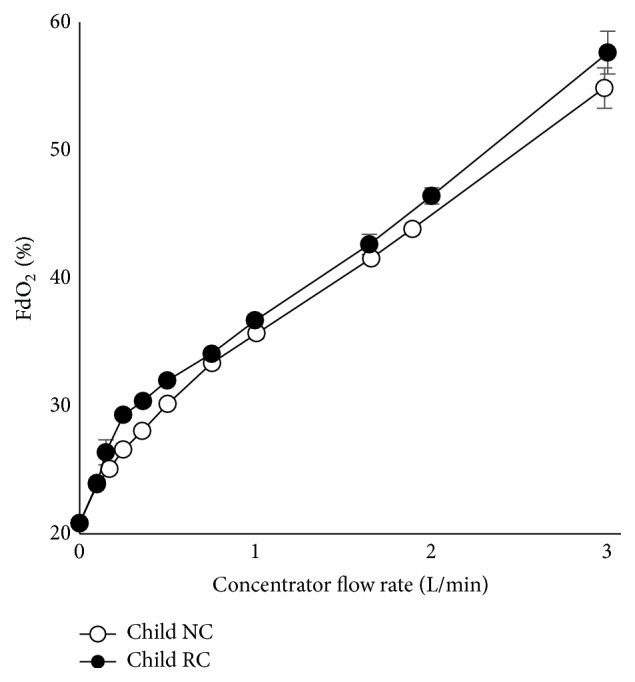
Performance test of RC and NC on the child lung model.

**Figure 7:**
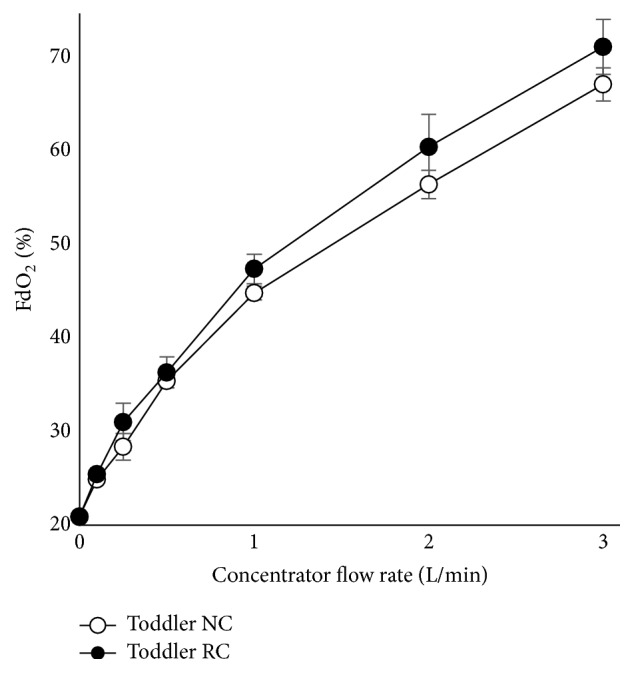
Performance test of RC and NC on the toddler lung model.

Using small child and toddler airway models with pediatric NC or modified RC, FdO_2_ values for the RC were higher than for the NC over the range of flows tested (Figures 6 and 7). Peak FdO_2_ values were high (greater than 50%), but this was expected given the relatively high-flow rates tested.

### 3.3. Oxygen Savings Ratio and Absolute Oxygen Flow Rate Savings

**Figure 8:**
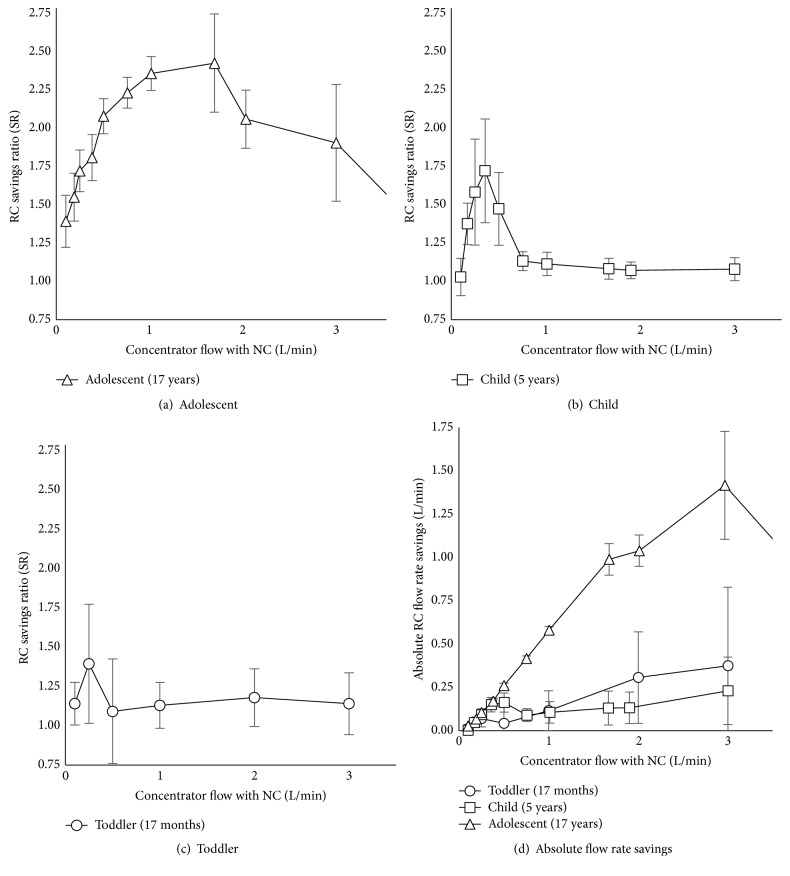
(a)–(c) Reservoir cannula (RC) oxygen savings ratio (SR) for three patient models and (d) absolute oxygen flow rate savings, compared to nasal cannula (NC) oxygen flow.

SR values for pediatric models are summarized in Figures 8(a)–8(c). Use of RC with all models exhibited increased savings compared with NC for flows between 0 and 0.5 L/min. The greatest savings (SR value) were observed with the adolescent model. Maximum SRs were 2.4 ± 0.3, 1.7 ± 0.3, and 1.4 ± 0.4 for adolescent, small child, and toddler models, respectively.

Absolute oxygen flow rate savings are shown in Figure 8(d). In general, oxygen savings improved with greater demand settings, such as with adolescents. AFS observed were as high as 1.4 ± 0.3, 0.2 ± 0.1, and 0.3 ± 0.3 L/min for adolescent, small child, and toddler models, respectively.

## 4. Discussion

We developed an* in vitro* method for testing RCs for pediatric applications and obtained high-precision FdO_2_ measurements. To the best of our knowledge, this is the first quantitative analysis of potential RC use for pediatric breathing parameters. Using this framework, we observed a range of oxygen savings when RCs were used instead of NCs in toddler, child, and adolescent breathing simulations (Figure 8). Overall savings observed were minimal for the toddler and child models, with total savings amounting to less than 0.3 L/min for the range of input flows tested. However, even this relatively small saving can amount to significant reductions in long-term oxygen usage: a flow reduction of 0.3 L/min over a 2-to-5-day treatment period would result in 860 to 2,160 fewer liters of oxygen per patient, or savings of about 15%, using the WHO requirements noted earlier.

As patient age increases, so does the tidal volume of each breath and the peak inspiratory flow rate. An increase in these two parameters could explain the improvement in performance seen in Figure 8. Larger tidal volumes and inspiratory flows may utilize reservoir gases more efficiently. Another explanation could be from longer inspiratory times (lower respiratory rate), giving more time per breath to fully consume reservoir gases. It should be noted again that the RCs were modified with smaller-bore pediatric NC prongs, which could also impact performance by increasing resistance of the tubing. Future work is required to develop a reservoir cannula that is optimized to pediatric prong sizes, inspiratory times, tidal volumes, and inspiratory flows, perhaps by using a more flexible or different volume reservoir.

The results of the FdO_2_ study in our adolescent model (Figure 5) were similar to that reported by the manufacturer for adult simulations [18]. Our adolescent model settings (see Table 1) varied only slightly from those of adult simulation lung model settings (respiration rate 16.5; tidal volume 500 mL; inspiration to expiration ratio 1 : 1) [18]. A dedicated pediatric reservoir with, for example, smaller reservoir volume and higher membrane compliance could be better suited for children and yield improved results.

### 4.1. Comparison with Other Oxygen Conservation Techniques

Continuous flow oxygen delivery via NC is a suboptimal form of oxygen delivery because inspiration often constitutes 33% or less of the breathing cycle [26], and some inspired gas remains unused in the anatomical dead space of the conducting airways [26]. Methods in addition to RCs have been studied for improving oxygen delivery efficiency via conservation. Pulsed or demand oxygen delivery devices can be used to conserve oxygen supplies by precisely delivering a bolus of oxygen during inspiration, the only time when oxygen is brought into the lungs. In adults, such devices reduce the delivered oxygen by as much as 86% to achieve the same FdO_2_, thereby allowing an oxygen cylinder to last up to 7 times longer than if oxygen were delivered continuously [27]. Portable oxygen concentrators often use pulse or demand sensing, allowing for lighter-weight and more power-efficient devices. However, they are relatively expensive and cannot provide flow to multiple patients. Furthermore, failure to sense inspiration can result in no oxygen flow.

Another oxygen conservation technique uses transtracheal or nasopharyngeal catheters that provide oxygen savings by bypassing some airway dead space [28–30]. However, these techniques are invasive and require humidification and monitoring to avoid serious complications from nasal secretion blockage. These problems make other methods of oxygen conservation unsuitable for many low-resource settings.

### 4.2. Future Work

Appropriately designed and validated RCs for effective use in children could be used with either cylinders or oxygen concentrators. Even with the relatively small flow savings observed in this model from 0.5 to 2 L/min, the logistical and cost burdens associated with oxygen cylinder refills could be reduced, and RCs could be advantageous with oxygen concentrators that have decreased power requirements when providing lower flow rates, especially when operating on battery power. RCs could be especially useful during peak pneumonia seasons, when the patient caseload exceeds the normal volume. During these times, the daily collective flow rate may exceed an oxygen concentrator's capacity for short periods due to seasonality, prevalence increases, and random effects [13]. During such peak events, additional RCs could be used in lieu of investment in more or larger concentrators and oxygen cylinders. However, this would require maintaining RCs in stock for use during these peak periods.

While an adult RC was used for our testing, an appropriately designed and affordable device optimized for pediatric patients will allow more accurate estimation of potential savings for pediatric applications. Designs using a more compliant reservoir material or different reservoir volume could be explored to improve oxygen savings. Various cannula prong sizes should be designed to accommodate the range of naris sizes exhibited by children. In addition, designs that increase the reusability of RCs could be advantageous, increasing their cost-effectiveness in comparison to NCs.

More importantly, the performance of such a device must be validated in clinical settings—and clinical assessment of safety and efficacy can be costly and time-consuming. Children and adults differ anatomically, in addition to differing in size [31], and this influences the methods and apparatuses appropriate for various clinical procedures, such as airway management during anesthesia [32]. Such differences could also influence measured oxygen savings. Given the wide range of savings observed in our study (Figure 8) and the variable nature of pediatric anatomy and breathing profiles, future studies should record both observed patient fit and oxygen savings.

Cost-effectiveness should also be evaluated in order for RCs to be proposed as a substitute for NCs. No gain in clinical effectiveness is anticipated with RC use over NC use because oxygen is delivered based on patient clinical status, which is best assessed by pulse oximetry. Therefore, efforts should be focused on reducing cost. Unfortunately, RCs are currently more expensive than standard NCs, with market price for the former at US$20 each and the latter at up to US$2 each. A thorough cost analysis (of both fixed and variable components) would be needed to determine whether the added cost associated with using RCs outweighs the cost of purchasing additional oxygen sources.

### 4.3. Study Limitations

RCs are currently only produced by one manufacturer in two form-factors for adults: the Oxymizer mustache-style (O-224, F-224) and Oxymizer pendant-style (P-224). This resulted in some limitations to our study.

We only tested cannulas (either with or without modification) to achieve roughly a 50% fit ratio to our airway models. Such gaps between the nares and the cannula RCs may not allow generation of sufficient negative pressure at the nasal prongs to draw gas from the reservoir, and different levels of fit may produce different results due to the physics of inhalation drawing oxygen from the reservoir. The availability of different cannula sizes for different-sized patients may affect the amount of savings in clinical practice. Variations in levels of mouth breathing could also result in lower oxygen savings [33].

Only the pendant-style RC was investigated because the relatively large reservoir can be placed away from the child's face, whereas a mustache-style RC would rest directly on the face. However, the mustache-style version could be explored if an apparatus that is fixed to the patient face is desired, as found in continuous positive airway pressure prongs. It is possible that the mustache cannula may perform better due to the closer proximity of the reservoir to the nose.

Another limitation was that the test lung equipment did not simulate consumption of oxygen from gas exchange. To minimize this limitation, the two-way valve was used to eliminate rebreathing of oxygen-rich exhaled air. Normal operation of the RC may use partial rebreathing into the reservoir, so these savings would not be accounted for in our study results. The test lung setup also had a fixed anatomical dead space volume of 200 mL, larger than an estimated range of 40 mL to 170 mL for the models tested, based on findings from another study [34]. Measurements were recorded after roughly 5 minutes when the system should have reached steady state and averaged over several breaths to minimize the effect of dead space on the results. Lung model estimates can deviate from patient results, so clinical validation of the study findings is necessary.

Finally, the parameters chosen for the patient models were based on reference values for healthy children to demonstrate proof-of-concept of the model. Future work with elevated respiratory rate should be performed as it is a common sign for pathologies involving hypoxemia, such as pneumonia and respiratory distress syndrome [35]. An increase in respiratory rate could potentially reduce the observed oxygen savings with RCs, but this could also be mitigated through developing a reservoir geometry that is appropriately optimized for this condition.

## 5. Conclusions 

RC testing demonstrated the potential for this technology to conserve oxygen in pediatric patients, but additional technical development and clinical validation are necessary. The findings from this study provide direction for future product development and clinical research.

## Figures and Tables

**Table 1:** Test lung settings used to simulate three healthy pediatric conditions.

Airway model	Respiratory rate (breaths/min)	Tidal volume (mL)	Inspiratory time (seconds)	Resistance (cm H_2_O/L/second)	Compliance (mL/cm H_2_O)	Inspiratory to expiratory ratio
Adolescent	15	400	1	5	100	1 : 2
17 years (80 kg) [25]						
Small child	20	100	0.85	15	20	1 : 2
5 years (20 kg) [24]						
Large infant/toddler	25	60	0.65	20	15	1 : 2
17 months (15 kg) [23]						
